# Microbiomics and metabolomics explored the characteristics of gut microbiota and metabolites in patients with aortic dissection

**DOI:** 10.3389/fcimb.2025.1677726

**Published:** 2025-10-08

**Authors:** Wenkun Liu, Qiu Lin, Huiying Zhong, Jing Liang, Binmei Liu, Yunnan Hu

**Affiliations:** ^1^ Department of Cardiology, Fujian Medical University Union Hospital, Fuzhou, China; ^2^ Fujian Cardiovascular Medicine Center, Fuzhou, China; ^3^ Fujian Institute of Coronary Artery Disease, Fuzhou, China; ^4^ Fujian Cardiovascular Research Center, Fuzhou, China; ^5^ Fujian Medical University Heart Center, Fuzhou, China; ^6^ Department of Clinical Laboratory, Fujian Medical University Union Hospital, Fuzhou, China; ^7^ Department of Cardiovascular Surgery, Fujian Medical University Union Hospital, Fuzhou, China; ^8^ Key Laboratory of Cardio-Thoracic Surgery (Fujian Medical University), Fuzhou, China

**Keywords:** aortic dissection, phenotype, gut microbiota, gut metabolites, metabolic pathway

## Abstract

Aortic dissection (AD) is a serious and frequently fatal condition with highly variable presentations, which increases the difficulty of diagnosis during the incubation period. The objective of this study was to reveal the influence of gut microbiota and metabolites on the occurrence and development of AD. In the present study, a total of 132 volunteers were recruited, but only 50 met the experimental requirements (including 25 health controls and 25 patients with AD). Patients with AD showed the high levels of systolic blood pressure (SBP) and diastolic blood pressure (DBP), accompanied with aortic dilation. High-throughput sequencing revealed a reduction in the abundance of beneficial bacteria (containing *Bifidobacterium* and [*Eubacterium*]_*eligens*_*group*) and an increase in harmful bacteria (containing *Desulfovibrio* and *Hungatella*) in patients with AD. In addition, untargeted metabolomic identified a total of 304 metabolites that were remarkably changes in AD patients, which major involved in alactose metabolism, caffeine metabolism, tyrosine metabolism, taurine and hypotaurine metabolism, ascorbate and aldarate metabolism, and butanoate metabolism. The above data elucidate that distinct gut microbiota and metabolites in AD patients, offering reliable information to building the prediction models of AD.

## 1 Introduction

Aortic dissection (AD) is a life-threatening rare disease that has a high mortality rate, influencing approximately 4-5 per 100,000 individuals in the USA (DePaolo et al., 2025). With changes in dietary structure and environment deterioration, the incidence of AD has been gradually increasing in China and is showing a trend toward younger age groups, which has placed a great burden on the country’s healthcare system (Chen et al., 2025b). AD is mainly manifested as a tear of the inner layer of the aorta, allowing blood to enter artery walls, resulting in separation between the inner and middle layers (Xu et al., 2025). Some studies have suggested that the occurrence of AD may be related to intense cell infiltration, inflammatory responses, oxidative stress, and destructive extracellular matrix remodeling (Hou et al., 2025). Once a dissection occurs, the mortality rate increases rapidly. At present, the regulation of blood pressure is the foundation of optimal medical management, and surgical repair depends on the anatomical location and related complications. Despite recent advances in acute management strategies and blood pressure control, AD remains related to high in-hospital mortality (Kimura et al., 2025). Consequently, it is important to develop methods to diagnosis AD accurately, which is help to provide timely treatment for AD patients.

The gut microbiota consists of trillions of microorganisms (including bacteria, fungi, viruses, and archaea) residing in the gastrointestinal trac, and it plays a crucial role in controlling nutritional metabolism enhancing immune system, suppressing pathogen invasion, and maintaining the intestinal barrier (Franklin et al., 2025). The composition of gut microbiota is influenced to a certain extent by factors such as diet, years, heredity, climate, and diseases (Zhang et al., 2025). Conversely, some studies have reported that an imbalance in gut microbiota can contribute to the occurrence of hypertension, hyperlipidemia, hyperglycemia, Alzheimer’s disease, heart failure, liver injury and other condition. The imbalance is mainly manifested as an obviously decreases in beneficial bacteria and a clearly increases in harmful bacteria abundance (Agus et al., 2018; Cai et al., 2022). For example, the abundance of *Bacteroides caecimuris*, *Veillonella parvula*, *Clostridium bolteae*, *Bacteroides xylanisolvens*, and *Ruminococcus gnavus* was found to be higher in patients with liver injury than in healthy individuals (Behary et al., 2021). Fecal microbiota transplantation have also confirmed that changes in the gut microbiota can replicate abnormal physiological parameters elevated serum glucose level, inflammatory responses, oxidative stress, and intestinal barrier injury (Mocanu et al., 2021). In addition, alterations in the gut microbiota lead to changes in gut metabolites. With the development of omics technologies, gut metabolites have been reported to obvious effects host health and are used in clinical diagnosis of certain diseases (Guo et al., 2025). A previous study displayed that gut microbiota-derived trimethylamine N-oxide and phenylacetylglutamine elevate the risk of cardiovascular disease by stimulating the inflammation and oxidative stress (Chen et al., 2025a). Although the role of gut microbiota and metabolites in improving host health has been widely demonstrated, their influence on the occurrence and development of AD remains unclear.

In this study, 132 volunteers were recruited from the Fujian Medical University Union Hospital (Fuzhou, China) from December 2023 to May 2025, but only 50 participants met the experimental requirements (Zhang et al., 2024). The objective of this study was to investigate the association between gut microbiota/metabolites and the occurrence of AD by high-throughput sequencing and untargeted metabolomics-based liquid chromatography-Orbitrap-tandem mass spectrometry (LC-Orbitrap-MS/MS), respectively. The data from this study provide valuable information for the clinical diagnosis of potential AD patients.

## 2 Materials and methods

### 2.1 Participant recruitment

**Figure 1:**
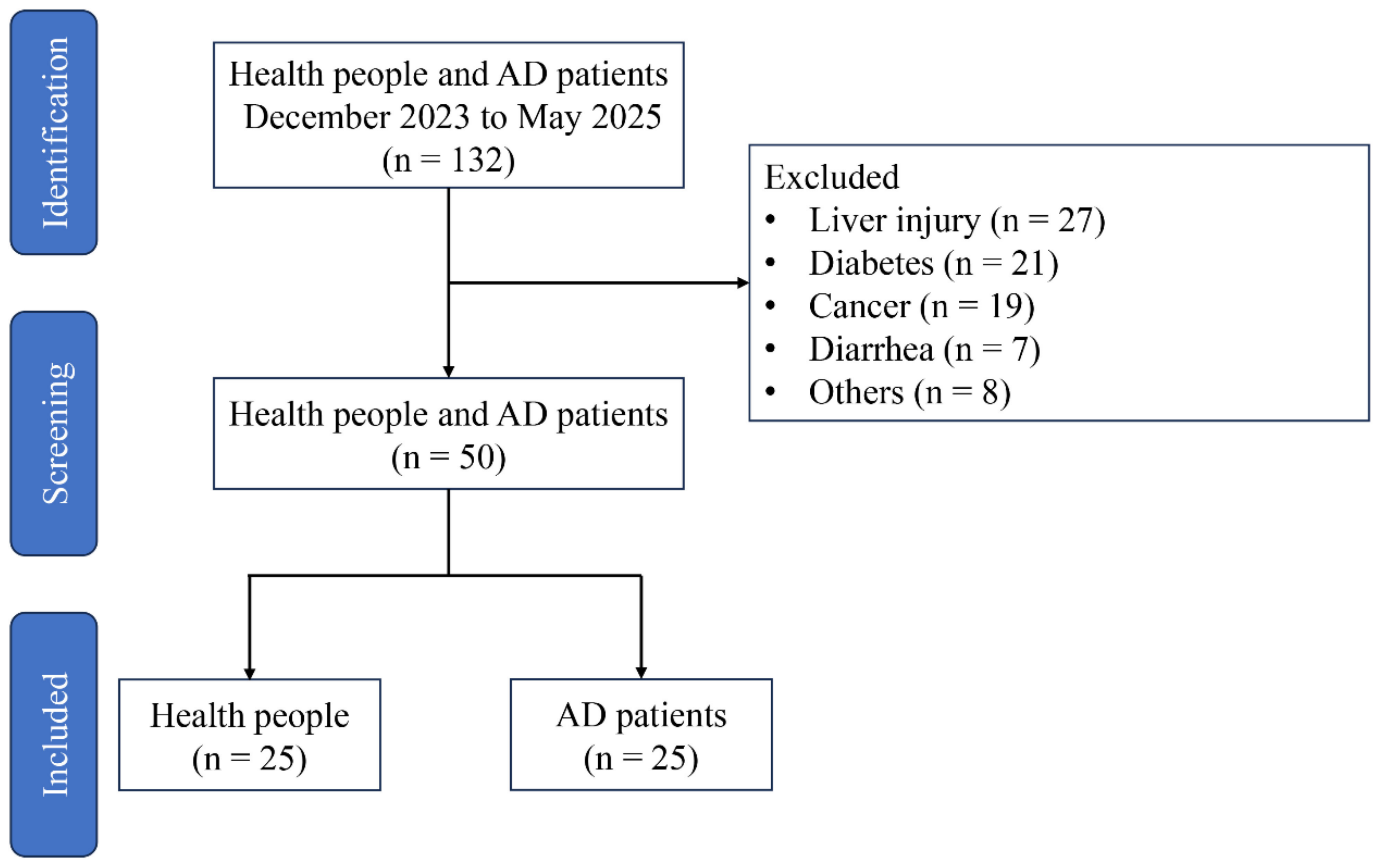
Study flow diagram.

Participant were recruited from the Fujian Medical University Union Hospital in Fuzhou, China between February 2023 and May 2025. The study was conducted in accordance with the Declaration of Helsinki, and approved by the Ethics Committee of Fujian Medical University Union Hospital (Approval No. 2023KJCX039). A total of 132 participants were recruited, but whom 50 met the inclusion criteria and were enrolled in the study (**Figure 1**). AD patients were excluded from the study based on the following criteria: congenital heart disease, recent surgical history, malignancy, systemic inflammatory disorders, intestinal ischemia, ongoing pregnancy, acute infectious diseases, absence of subsequent surgical repair, lack of signed informed consent, or insufficient baseline data. In addition, participant had not used antibiotics and used tobacco in the 12 weeks prior. The diagnosis of AD was proven at surgery and computed tomography. Fecal samples from AD patient and healthy people were collected after obtaining informed consent from the subjects. All sample were quick-frozen using liquid nitrogen, and then stored at -80°C for further analysis.

### 2.2 Data collection

Detail information of the participants, including both AD patients and healthy controls, was collected and recorded. Data included age, gender, body mass index (BMI), blood and blood pressure, among others. The white blood cell (WBC) and RBC in the blood were measuring by a Beckman Coulter analyzer. In addition, blood sample were collected, and placed at the room temperature for 2 h. After centrifugation (4000 ×g, 15 min, 25°C), the supernatant were collected to measured the serum alanine aminotransferase (ALT), aspartate transaminase (AST), alkaline phosphatase (ALP), triglyceride (TG), and glucose (GLU) levels. All participants were classified into two groups based on the clinical diagnosis: the AD group (patients with AD) and the NC group (healthy individuals).

### 2.3 Thoracoabdominal aortic computed tomographic angiography

Participants are positioned supine on the computed tomography (CT) scanner table, and an intravenous catheter is placed, typically in an antecubital vein. A bolus of iodinated contrast medium is injected intravenously at a high flow rate using an automated power injector. A helical (spiral) CT scan is performed from the thoracic inlet (above the aortic arch) down to the common femoral arteries, synchronized with the arrival of the contrast bolus in the aorta.

### 2.4 Echocardiography analysis

Participants were placed in the left lateral decubitus position with the chest fully exposed for examination. The operator held a phased-array transducer and sequentially places it at standard acoustic window, including the parasternal, apical, subxiphoid, and suprasternal regions. The system acquired and recorded standard two-dimensional echocardiographic views, M-mode echocardiograms, and Doppler spectra of the heart. Image quality was optimized by adjusting transducer parameters including angle, depth, and gain.

### 2.5 Gut microbiota analysis

Fresh fecal samples were quick-frozen using liquid nitrogen and transported to Majorbio Biotechnology Corporation (Shanghai, China). Fecal microbial genomic DNA was obtained using the E.Z.N.A. Fecal DNA Kit (Omega Bio-tek, GA, USA). The V3-V4 region of the 16S rRNA gene was PCR-amplified with the primers 338F (5′-ACTCCTACGGGAGGCAGCA-3′) and 806R (5′-GGACTACHVGGGTATCTAAT-3′). Amplification products were detected by 2.0% agarose gel electrophoresis, and the target bands were recycled and collected using the AxyPrep PCR Cleanup Kit (Thermo Fisher, CA, USA). The purified products were used to establish libraries, and were sequenced on the Illumina MiSeq platform (Illumina, San Diego, USA).

Raw sequencing data, which have been deposited in the NCBI database, were processed and filtered using QIIME2 to obtain high-quality reads. These sequences were then clustered into operational taxonomic units. Alpha diversity indices (Chao1, Observed, Shannon, and Simpson indices) of the gut microbiota were analyzed using appropriate bioinformatic tools (e.g., Xshell 7.0). Beta diversity was carried out by principal component analysis (PCoA). Bacterial taxa showing significant differences between the control and AD groups were identified using extended error bar plots based on Welch’s t-test.

### 2.6 LC-Orbitrap-MS/MS analysis

Fresh fecal samples were quick-frozen using liquid nitrogen, and subsequently freeze-dried using a freeze dryer. Each sample (200 mg) and 0.8 mL of extraction solution (acetonitrile:methanol = 4:1), and incubated at room temperature for 30 min. After vortexing for 30 s, the mixture was centrifuged (14000 ×g, 15 min, 4°C), and 0.4 mL of supernatant were collected. The supernatant was then freeze-dried using a vacuum centrifugal concentrator and reconstituted in 0.2 mL of the extraction solution. The resulting solution was filtered through a 0.22 μm microfilter. A quality control (QC) sample was prepared by pooling equal volumes from each sample. Finally, metabolites in the fecal samples were analyzed using LC-Orbitrap-MS/MS (Thermo Fisher Scientific, Waltham, MA, USA).

The raw data were processed by Compound Discoverer 3.8 for peak detection, alignment, and normalization. The whole structure of fecal metabolites was achieved using R software, potential markers were identified using Compound Discoverer 3.8 by matching against the HMDB, METLIN, Massbank, and SMPDB database. Pathway enrichment analysis was carried out using MetaboAnalyst 6.0 according to the difference metabolites.

### 2.7 Statistical analysis

The data were presented as mean ± SD, the differences between the control and AD groups were analyzed using SPSS 22.0.

## 3 Results

### 3.1 Identification of AD

**Figure 2:**
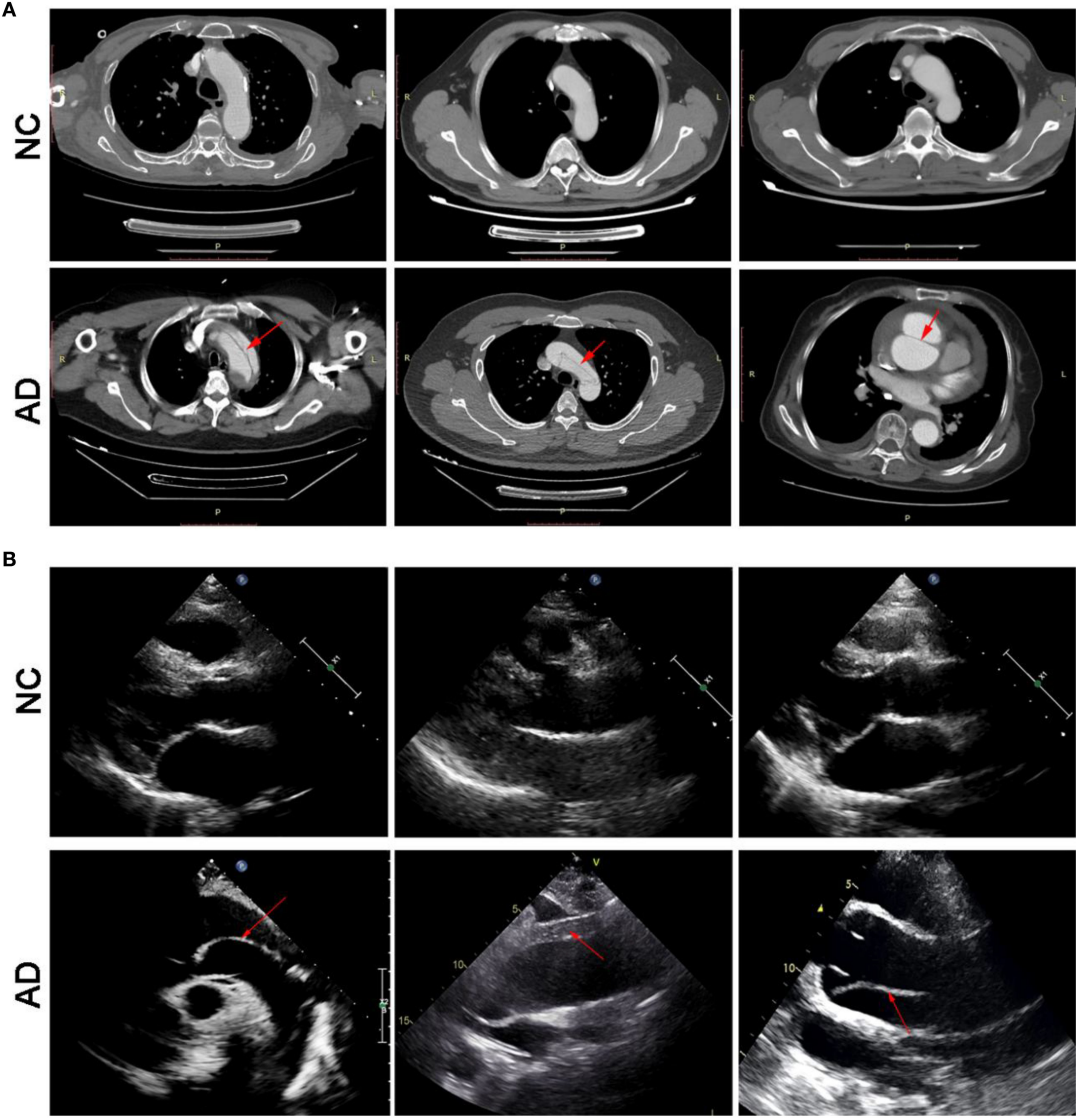
Transverse view of aortic computed tomography angiography **(A)**, and transthoracic echocardiography **(B)**.

On enhanced computed tomography images, a linear low-density shadow (representing an intimal flap) can be visualized within the aortic lumen in patients with AD, which separates the aorta into true and false lumina (**Figure 2A**). In addition, the result of transthoracic echocardiography showed that aortic lumen of AD patients was widened, along with torn intimal echo in the lumen. These appeared as linear or strip-like structures that swayed throughout the cardiac cycle, dividing the lumen into true and false channels (**Figure 2B**). Notably, there was no occurrence of intestinal ischemia in any patient with AD (**Supplementary Figure S1**). However, these phenomena have not been observed in the NC group.

### 3.2 Patient characteristics

As illustrated in **Table 1**, a total of 50 volunteers were recruited in the present research, comprising 25 healthy individuals (13 males and 12 females) and 25 patients with AD (18 males and 7 females). No remarkable differences were observed between the NC and AD groups in terms of BMI (24.71 ± 3.31 vs 26.12 ± 3.86), serum ALT (18.16 ± 8.51 vs 22.44 ± 15.40), serum AST (21.32 ± 5.98 vs 21.12 ± 12.49), serum ALP (70.12 ± 15.82 vs 68.32 ± 20.22), serum TG (3.95 ± 14.18 vs 1.39 ± 0.66) and glucose (5.11 ± 0.47 vs 5.65 ± 1.43) levels (*p* > 0.05), suggesting that the occurrence of AD is not directly related to obesity, liver function injury, dyslipidemia, and hyperglycemia. Notably, both systolic blood pressure (163.52 ± 19.39 vs 118.44 ± 9.14, SBP) and diastolic blood pressure (89.60 ± 20.64 vs 76.80 ± 7.88, DBP) in patients with AD were remarkable higher than that in health people (*p* < 0.01), indicating that the higher DBP and SBP levels may elevate the risk of AD. Moreover, patient with AD exhibited obvious higher levels of serum WBC and PLT compared with that in health people (*p* < 0.01).

**Table 1:** Descriptive statistics of the study participants.

Characteristic	NC (n = 25)	AD (N = 25)	*p*-value
Age (years)	58.00 ± 11.93	52.52 ± 11.78	0.10
Gender (m/f)	13/12	18/7	–
BMI (m^2^/kg)	24.71 ± 3.31	26.12 ± 3.86	0.17
SBP (mmHg)	118.44 ± 9.14	163.52 ± 19.39	3×10^-12^
DBP (mmHg)	76.80 ± 7.88	89.60 ± 20.64	0.0069
WBC (10^9^/L)	6.31 ± 1.33	8.76 ± 3.59	0.0033
PLT (10^9^/L)	235.20 ± 37.67	206.52 ± 45.53	0.019
NEU (%)	61.36 ± 4.41	71.11 ± 15.40	0.0051
ALT (U/L)	18.16 ± 8.51	22.44 ± 15.40	0.23
AST (U/L)	21.32 ± 5.98	21.12 ± 12.49	0.94
ALP (U/L)	70.12 ± 15.82	68.32 ± 20.22	0.73
TG (mmol/L))	3.95 ± 14.18	1.39 ± 0.66	0.38
GLU (mmol/L)	5.11 ± 0.47	5.65 ± 1.43	0.08

ALP, alkaline phosphatase; ALT, alanine aminotransferase; AST, aspartate transaminase; BMI, body mass index; DBP, diastolic blood pressure; GLU, glucose; NEU, neutrophil count; PLT, platelets; SBP, systolic blood pressure; TG, triglyceride; WBC, white blood cell.

### 3.3 Alteration of gut microbiota in patients with AD

**Figure 3:**
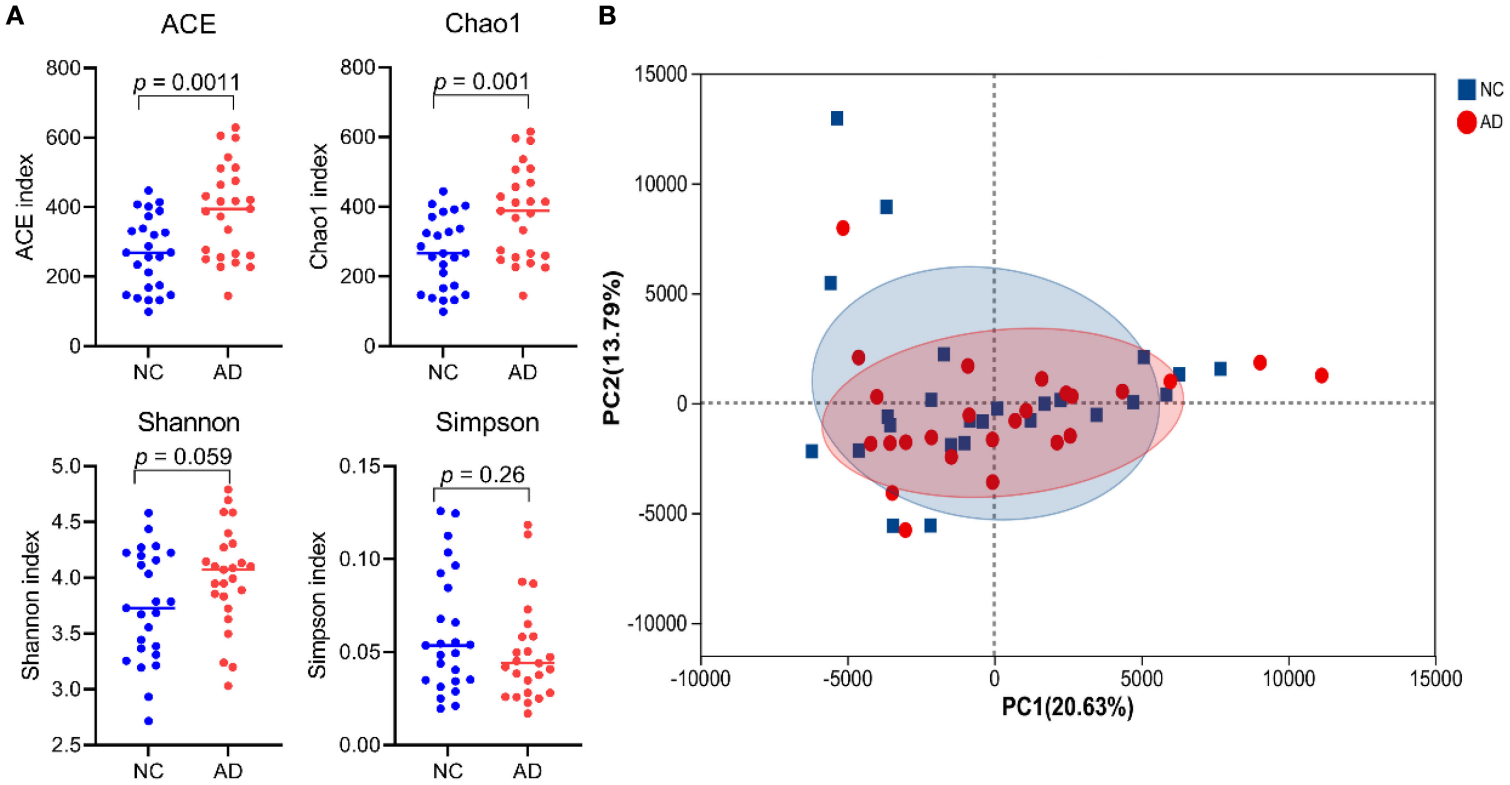
The alpha- **(A)** and beta-diversity **(B)** of gut microbiota in health people and AD patients.

To explore the association between gut microbiota and the occurrence of AD, the 16S rRNA sequencing technology based on the V3-V4 hypervariable region was performed in both healthy individuals and patients with AD. Alpha-diversity (including ACE, Chao1, Shannon, and Simpson indexes) was widely applied to evaluate the richness and diversity of bacterial species. As shown in **Figure 3A**, the ACE (386.89 ± 135.22 vs 267.11 ± 105.47) and Chao indexes (382.46 ± 132.22 vs 264.69 ± 103.80) of gut microbiota in the AD group were remarkably higher than that in the NC group (*p* < 0.01), suggesting that AD patients induced changes in richness and diversity in the gut microbiota. There was no significant difference in Shannon (3.74 ± 0.50 vs 4.00 ± 0.45) and Simpson indexes (0.06 ± 0.03 vs 0.05 ± 0.03) between the NC and AD group (*p* > 0.05), but Simpson indexes in the AD group displayed an obvious decline compared with the NC group. In addition, beta diversity analysis based on PCA using Bray-Curtis distance was applied to reveal differences in the whole composition of gut microbiota between the NC and AD group (**Figure 3B**). In the PCA plot score, the first and second principal component (PC1 and PC2) accounted for 20.63% and 13.79% of the total variance, respectively. The result of PCA exhibited no obvious separation in gut microbiota composition between the NC and AD groups.

### 3.4 Screening for key microbial phylotypes

**Figure 4:**
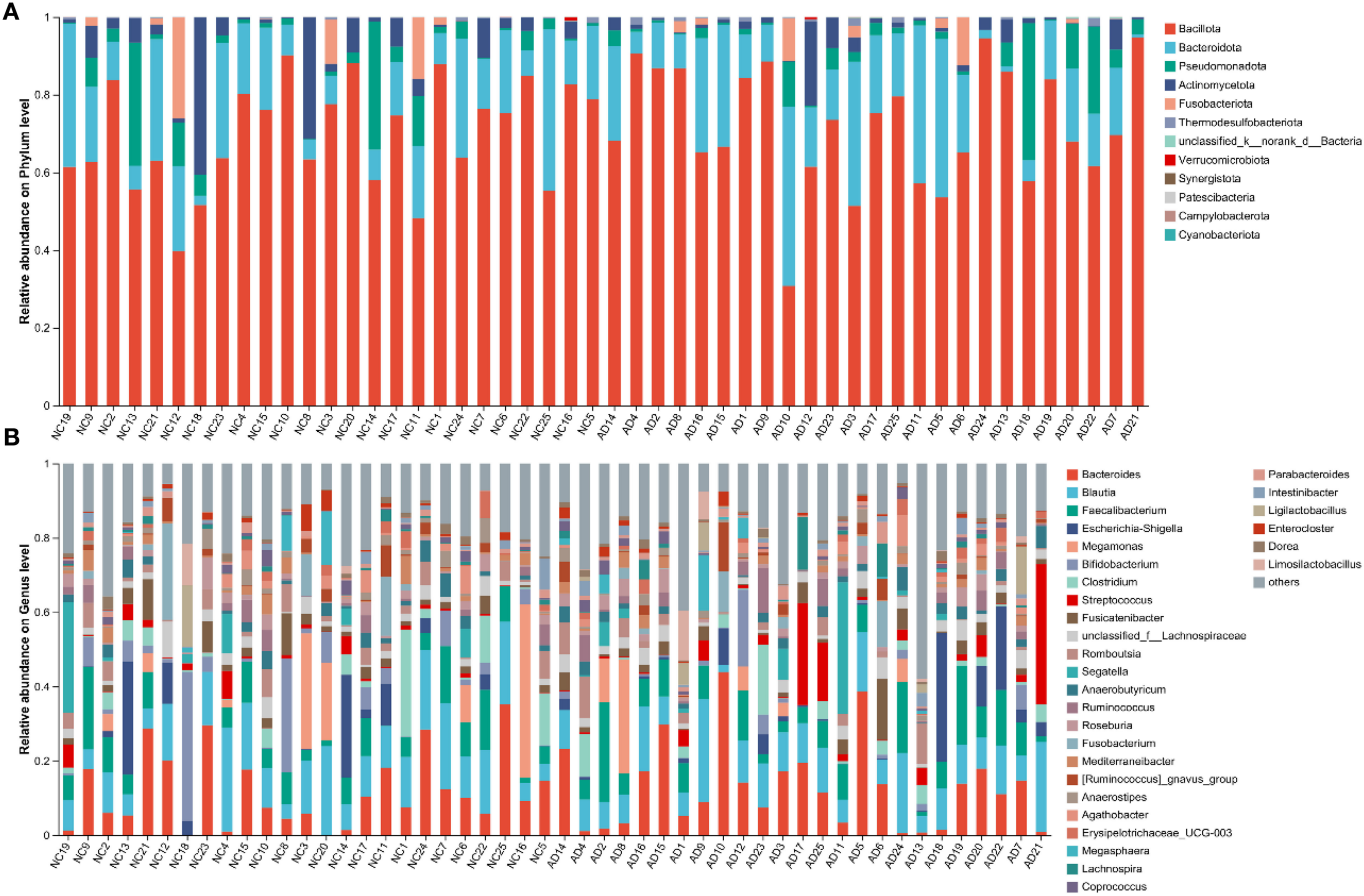
Proportion of bacterial in feces from health people and AD patients. **(A)** Phylum and **(B)** genus.

To deeply characterize the gut microbiota responsible for the occurrence of AD, the relative abundance of gut microbiota at different taxonomic levels between the NC and AD groups. At the phylum level, Bacillota, Bacteroidota, Pseudomonadota, Actinomycetota, and Fusobacteriota were the most abundant phyla in both groups, with Bacillota and Bacteroidota being particularly predominant (**Figure 4A**). Notably, the relative abundance of Bacillota and Bacteroidota accounted for more than 75% of the gut microbiota in the NC and AD groups. At the genus level, the gut microbiota in the NC and AD groups was major consisted of *Bacteroides*, *Blautia*, *Faecalibacterium* e*scherichia*-*Shigella*, *Megamonas*, *Bifidobacterium*, *Clostridium*, Streptococcus, Fusicatenibacter, unclassified_f_Lachnospiraceae, Romboutsia, Segatella, *Anaerobutyricum*, *Ruminococcus*, *Roseburia*, *Fusobacterium*, *Mediterraneibacter*, [*Ruminococcus*]_*gnavus*_*group*, *Anaerostipes*, *Agathobacter* e*rysipelotrichaceae*_*UCG*-003, *Megasphaera*, and *Lachnospira*, which accounted for more than 70% (**Figure 4B**).

**Figure 5:**
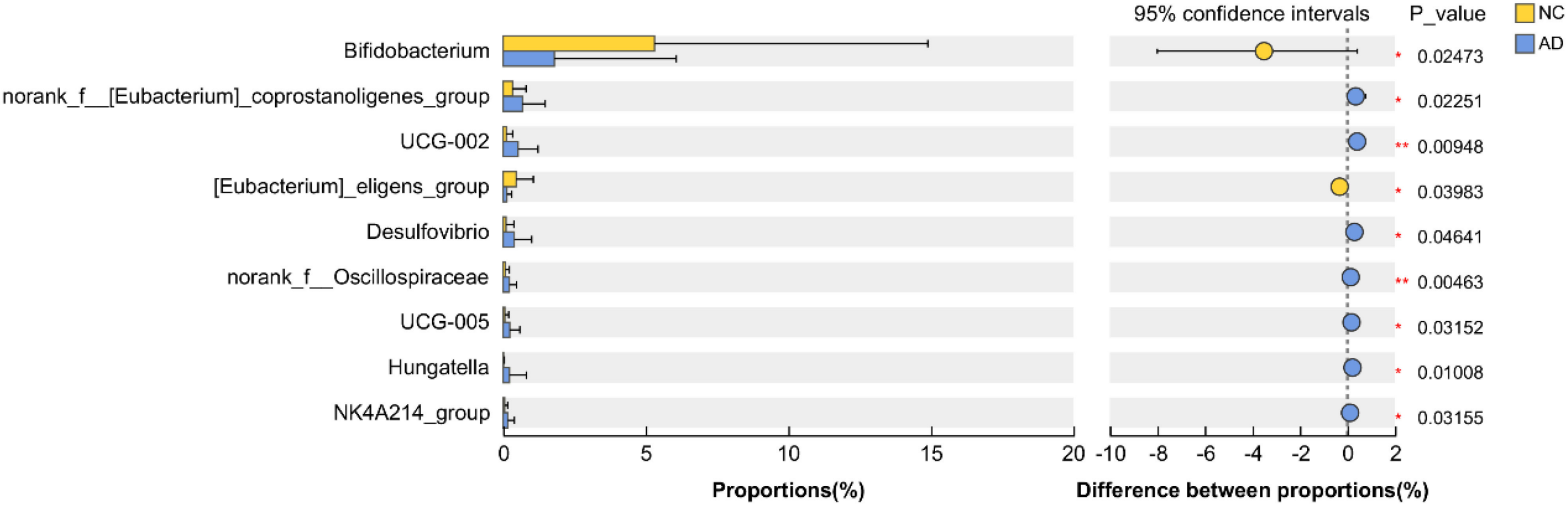
Extended error bar analysis of gut microbiota was implemented based on Welch’s t-test.

Interestingly, the relative abundance of *Bifidobacterium* and [*Eubacterium*]_*eligens*_*group* in the AD group were significantly reduced compared with the NC group (*p* < 0.05), but the relative abundance of *norank*_f_[*Eubacterium*]_*coprostanoligenes*_*group*, UCG-002, *Desulfovibrio*, *norank*_f_*Oscillospiraceae*, UCG-005, *Hungatella*, and NK4A214_group were significantly increased (*p* < 0.05), indicating AD markedly affected the gut microbiota composition (**Figure 5**).

### 3.5 Alteration of gut metabolites in patients with AD

**Figure 6:**
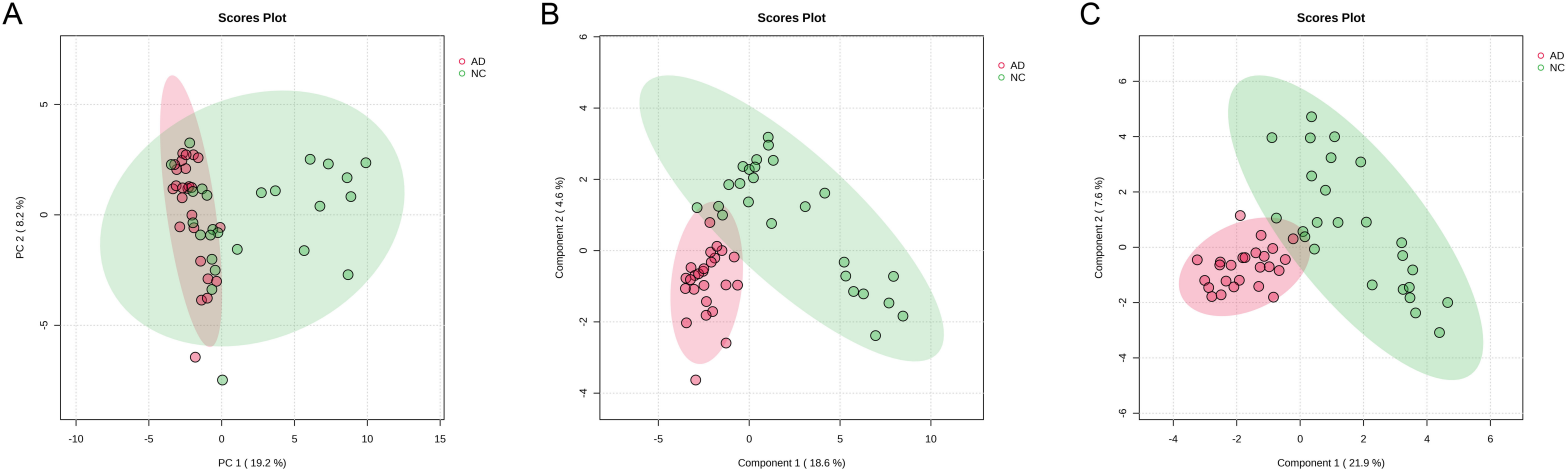
The change in gut metabolites in health people and AD patients. **(A)** PCA, **(B)** PLS-DA, and **(C)** sPLS-DA.

Alterations in the gut microbiota directly influence gut metabolic profiles, the gut metabolic profiles of both healthy individuals and patients with AD were detected using untargeted metabolomics based on LC-Orbitrap-MS/MS. In PCA plot score, samples from the NC group were distributed across all four quadrants, whereas those from the AD group were predominantly located in the second and third quadrants elaborating a distinct shift in the metabolic profile of fecal samples in AD patients (**Figure 6A**). Due to the limited discriminatory power of PCA, PLS-DA was applied to further characterize the overall characteristics of fecal metabolites (**Figure 6B**). The result of PLS-DA analysis displayed that an obvious separation between the NC and AD groups, indicating that significant alterations in fecal metabolites from patients with AD. This finding was further supported by the results of SPLS-DA (**Figure 6C**).

### 3.6 Screening for key gut metabolites

**Figure 7:**
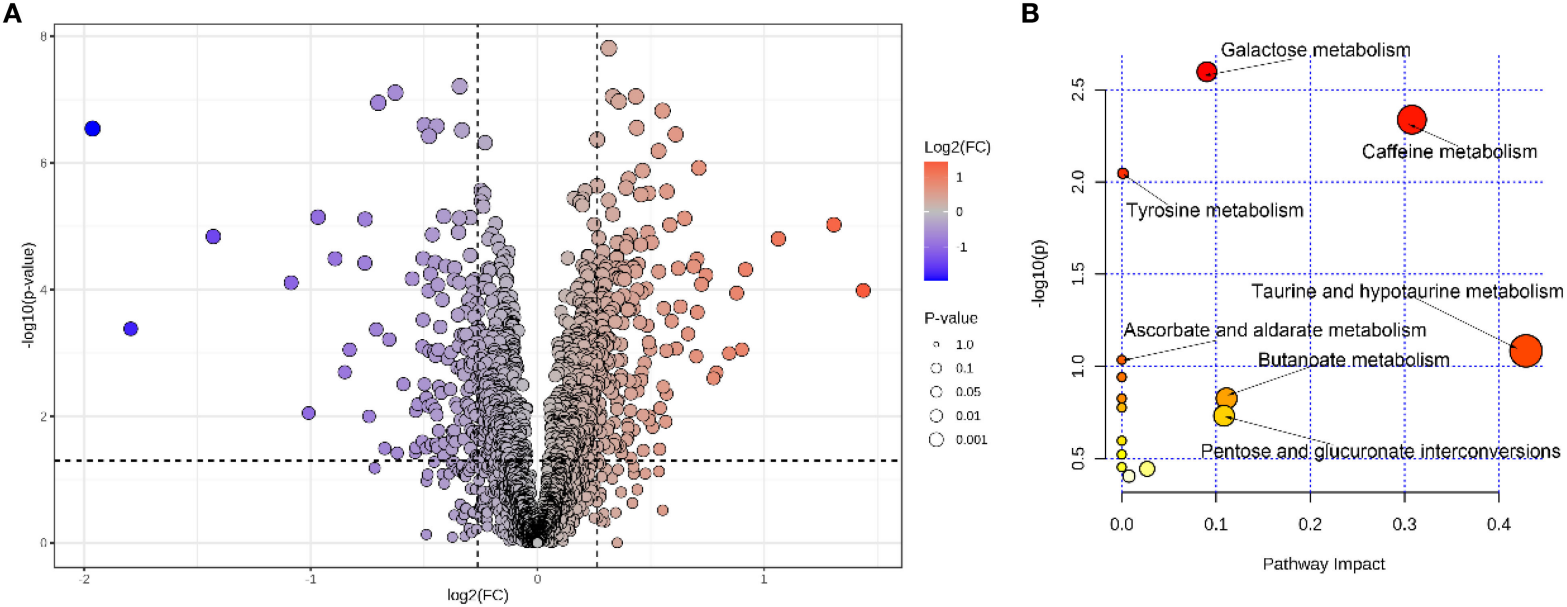
The volcano plot for gut metabolites between the NC and AD groups **(A)**, analysis of key metabolic pathways **(B)**.

Key gut metabolites in patients with AD were screened using volcano plot based on the criteria of VIP >1.0, FC > 1.2, and *p* < 0.05. As presented in **Figure 7A**, the results of volcano plot exhibited a total of 304 key metabolites that were altered in AD patients compared to healthy individuals, including the down-regulation of 120 key metabolites (including adenylosuccinic acid, 3’-deoxyderhamnosylmaysin, apigenin rubiadinprimeveroside, multiflorin B, galactaric acid, gentiopicrin, 5-methoxysalicylic acid sulfate, parishin C, apigenin 7-sulfate, diferuloyl putrescine, genistein 7-sulfate, (-)-epigallocatechin 3-glucuronide, 4-hydroxytriazolam, codeine-6-glucuronide, ochromycinone, plumieride, clethodim, maltotetraose, malonylgenistin, isoschaftoside, demethoxycentaureidin 7-O-rutinoside, apigenin 7-galacturonide, phloretin xylosyl-galactoside eriocitrin, taraxacoside, baicalin, stachyose, glycogen, 5-hydroxyindoleacetaldehyde, ophthalmic acid, homovanillic acid sulfate, N,N-dimethylguanosine, 3,4-dihydroxybenzenesulfonic acid, raffinose, cholesterol glucuronide, cinnamoylglycine, 3-methyluridine, cloranolol, spionoside B, procyanidin B1, 2-hydroxyfelbamate, 3’-benzoylsalicin, dicoumaroyl spermidine, physapubescin, lactofen, fexofenadine, kelampayoside a, lactarorufin B, N-lactoylleucine, implitapide, flavanone B, taurine, temazepam glucuronide, tryptophyl-glutamate, manninotriose, 3-sulfobenzoic acid, oxazepam glucuronide, ponasterone A, tuberonoid A, temocapril etc.) and the up-regulation of 184 key metabolites (including 2-methylhippuric acid, L-rhamnulose, altenusin, 3-pyridinol, andrographolide, halometasone, 8-angeloylegelolide, melatonin glucuronide, cucurbic acid, gentisic acid, daumone, baptifoline, stercobilin, 3,5-dimethoxycinnamic acid, acetoin, N-palmitoyl cysteine, lamiidoside, triacanthine, penciclovir, sobetirome, mimosine, prunasin, 3-methylorsellinic acid, megestrol esculetin, lysyltryptophan, dihydroresveratrol 3-glucuronide, methylisopelletierine, chaetoxanthone b, beraprost, (Z)-3-hexenal, deoxyelephantopin, cyperine, leucyl-tryptophan, vanillylmandelic acid, absindiol, topaquinone, quinolinic acid, cartorimine, 2-methylbenzoic acid, 3,4,5,6-tetrahydrohippuric acid, 4-hydroxyretinoic acid, dacinostat, 1-methylxanthine, acetoacetic acid, 4,5-dihydroniveusin A ethylmalonic acid, nopalinic acid, 1,3-dimethyluric acid, teloxantrone, 4’,5,8-trihydroxyflavanone, dihomolinoleic acid, momilactone A, coniferyl alcohol, alpha-zearalenol, vulgarole, 4-ethylphenol, rhamnose, tilisolol, 2-methylpyridin-3-ol, glucose butyrate, isokobusone, 5-hydroxyflavone, glyvenol, demethylvestitol, 2-acetyl-1-ethylpyrrole, dihydroorotic acid, lupinic acid, pinolidoxin, macrophorin B, tyrosyl-phenylalanine, indole-3-methyl acetate, cysteinyl-methionine, peliglitazar, myrianthic acid, atorvastatin tyrosyl-leucine, cerasinone, phenylalanyltyrosine, sedanonic acid, variabilin, acetylstrophanthidin, nepetalactam, obtustyrene, lithocholic acid etc.).

In order to further explore the fecal metabolic pathways associated with the occurrence and progression of AD, pathway analysis based on the KEGG database was implemented using MetaboAnalyst 6.0. After enrichment analysis, 304 key gut metabolites were mapped in 15 pathways, including galactose metabolism, caffeine metabolism, tyrosine metabolism, taurine and hypotaurine metabolism, ascorbate and aldarate metabolism, butanoate metabolism, pentose and glucuronate interconversions, and so on (**Figure 7B**).

## 4 Discussion

AD is a potentially fatal aortic event and a critical global health challenge. According to a previous study, the occurrence of AD is obvious related to gender, heredity, hypertension, obstructive sleep apnea, and the existence of a bicuspid aortic valve (Kahraman and Binicier, 2025). The mortality rate of patients with AD more than forty percent if effective treatment is not received in the first 24 h, and it increases rapidly to ninety percent under three months (McCarthy and Burke, 2025). Due to limited resources and diagnostic methods, many AD patients do not receive timely treatment shortly after onset. With advances of omics technologies, significant progress has been made in the diagnosis of various diseases. However, omics approaches have not yet been widely applied in the clinical diagnosis of AD. Therefore, we hypothesized that applying omics technology could improve early detection of AD before overt manifestation. In the present study, we characterized the gut microbiota and metabolomic profiles of AD patients and healthy individuals using high-throughput sequencing and untargeted metabolomics to identify key microbial phylotypes and metabolites associated with AD.

Blood pressure refers to the pressure exerted by circulating blood on the walls of blood vessels and is strongly associated with host health (Mark, 2025). In clinical practice, systolic blood pressure (SBP) in healthy individuals is generally below 120 mm Hg, and the diastolic blood pressure (DBP) below 80 mm Hg. In this study, both SBP and DBP levels of AD patients were obvious higher than the normal range, indicating that AD is commonly accompanied by hypertension, which is in agreement with a previous study (Shi et al., 2025). Therefore, the reduction in the SBP and DBP level may help suppress the occurrence of AD. In addition, ALT, AST, and ALP serve as essential parameters for detecting liver function. When the injury of liver function, AST and ALT are released from hepatocytes into the bloodstream, resulted to the increases in serum transaminases (Guo et al., 2021). ALP activity, which is linked to systemic inflammation, reflects the extent of liver damage to some extent (Guo et al., 2025). In the present study, no obvious differences in serum ALT, AST, and ALP levels between the NC and AD groups, suggesting that AD patients did not exhibit disease-related liver injury.

Accumulating evidence suggests a strong association between gut microbiota and certain types of disease-related cardiac injury, highlighting its role in mediating microbial interactions with drugs for the improvement of heart disease (Wu et al., 2025). Therefore, gut microbiota composition in health people and AD patients was detected using high-throughput sequencing. The ACE and Chao1 indexes directly reflect gut microbial richness, and high level of these indices are commonly associated with healthy populations. In this study, AD patients exhibited the high value of ACE and Chao1 indexes, which because of a “pathological compensation” or microbial community rearrangement rather than a beneficial restoration (Jiao et al., 2025).

The phyla Bacillota (which includes Gram-positive bacteria) and Bacteroidetes phylum (which includes Gram-negative bacteria) are extensively existed in the gut, and their ratio reflects the host’s glucolipid metabolism to a certain extent (Tan et al., 2024). In this study, no obvious differences were observed in the abundances of Bacillota and Bacteroidota between the NC and AD groups, indicating that disorder glucolipid metabolism did not induce the occurrence of AD. AD patients exhibited increases in Patescibacteria, Thermodesulfobacteriota, and a decrease in Fusobacteriota. Among them, Patescibacteria is a candidate phylum radiation that is highly abundant in the gut of patients with hepatocellular carcinoma, as it promotes the accumulation of reactive oxygen species and enhances glucose uptake in cancer cells (Huang et al., 2022). A high abundance of Thermodesulfobacteriota has been reported in Parkinson’s disease, this phylum has the ability to promote histamine production, which can damage both the nervous and immune systems (Proano et al., 2023).

*Bifidobacterium* is a major genus of gut bacteria, widely reported to possess various physiological functions, such as improving glucolipid metabolism, suppressing inflammatory responses, and alleviating oxidative stress. It inhibits the growth of harmful bacteria by producing short-chain fatty acids and competing for nutrients (Gao et al., 2023). *Bifidobacterium* has also been shown to protect against organ injury by reducing inflammation enhancing antioxidant enzyme activity, and modulating gut microbiota composition (Li et al., 2023). Our previous study demonstrated that oral administration of *B. pseudolongum* CCFM1253 effectively alleviated inflammation and oxidative stress by regulating the NF-κB and Nrf2 pathways, respectively (Guo et al., 2023). In addition, *[Eubacterium]_eligens_group* is a common short-chain fatty acid-producing bacterium that helps reduce the risk of heart diseases by lowering blood pressure, ameliorating glucolipid homeostasis, maintaining the integrity of the intestinal barrier, and regulating the immune system (Nogal et al., 2021). In the present study, the abundance of *Bifidobacterium* and *[Eubacterium]_eligens_group* in the AD group were remarkably reduced compared with the NC group, while the abundance of *Desulfovibrio* and *Hungatella* were remarkably increased. *Desulfovibrio* is a sulfate-reducing bacterium primarily found in the human gut and vaginal tract, which has been confirmed to promote the development of atherosclerosis by destroying the intestinal barrier and up-regulating the LR4/NF-κB pathway (Zhang et al., 2023b). A high abundance of *Desulfovibrio* aggravates dextran sulfate sodium-induced colitis by accelerating the secretion of IL-1β and TNF-α (Huang et al., 2024). *Hungatella* is an anaerobic bacterium originally isolated from the feces of patients with acute appendicitis, which elevated the 5-fluorouracil resistance of colorectal cancer cells by regulating the CDX2/β-catenin pathway (Huang et al., 2023). Therefore, we speculate that a reduction in harmful bacteria (*Desulfovibrio* and *Hungatella*) and an increase of beneficial bacteria (*Bifidobacterium* and *[Eubacterium]_eligens_group*) may help suppress the occurrence of AD. However, Gut microbiota alterations observed in patients with AD may be influenced by several confounding factors. Firstly, involvement of the superior mesenteric artery and consequent intestinal ischemia could significantly alter the gut microenvironment and microbial composition (Yesitayi et al., 2025). Secondly, the profound systemic stress and inflammatory response characteristic of the acute phase of AD may also exert substantial effects on the gut microbiota (Liu et al., 2025; Tian et al., 2022). Furthermore, the gut microbiota constitutes a dynamic ecosystem, shaped by a multitude of factors such as diet, age, genetics, antibiotics environment, and lifestyle. In the present study, congenital heart disease, recent surgical history, malignancy, systemic inflammatory disorders, intestinal ischemia, ongoing pregnancy, acute infectious diseases, used antibiotics and used tobacco were excluded to reduce their effect on the gut microbiota composition, but diet environment, and lifestyle represent important limitations of our study and should be considered when interpreting the results.

It is widely accepted that changes in gut microbiota directly effect the composition of gut metabolites and play the important role in the gut-heart axis (Chakraborty et al., 2024). As a result, we speculate that gut metabolites play the most important role in the occurrence and development of AD. In this study, the concentration of adenylosuccinic acid, galactaric acid, gentiopicrin, apigenin 7-sulfate, plumieride, maltotetraose, and isoschaftoside in the AD group were remarkably reduced compared with the NC group. Adenylosuccinic acid is a small molecule metabolite that enhances the activity of antioxidant enzymes by stimulating the expression of Nrf2 (Timpani et al., 2023). Galactaric acid (also known as mucic acid) is widely used in skincare products because of its strongly anti-aging properties (Kuivanen et al., 2016). Gentiopicrin, an O-glycosyl compound, is a naturally occurring metabolite that exhibits a wide range of pharmacological activities, including anti-apoptotic, hypolipidemic, hypoglycemic, hypotensive, anti-inflammatory, antioxidant, anticancer, and anti-fibrotic effects (Gao et al., 2025). For example, gentiopicrin supplement suppresses inflammation and oxidative stress by down-regulating the HF-κb/Iκ-Bα pathway and up-regulating the PPARα/Nrf2 pathway, respectively (Zhang et al., 2023a). Apigenin 7-sulfate belong to the flavone class and has been reported to inhibit myocardial apoptosis and inflammatory responses by down-regulating the SphK1/S1P pathway, which is beneficial for prevent lipopolysaccharide-induced heart injury (Zhang et al., 2015). Plumieride is an iridoid that has been widely used as anti-fungal and anti-inflammatory component, and is help to reduce the accumulation of lipid hydroperoxides and elevates superoxide dismutase activity (Boeing et al., 2018). Maltotetraose consists of four glucose units connected by α-1,4-glycosidic bond and functions as an inhibitor of TNF-α-induced ICAM-1 expression, thereby suppressing inflammatory responses (Shin et al., 2016). Isoschaftoside, a flavonoid, inhibits M1 polarization of macrophage cells and reduces TNF-α production in lipopolysaccharide-treated mice (Abe, 2025). However, the concentration of 2-methylhippuric acid, 3-pyridinol, stercobilin, sobetirome, vanillylmandelic acid, quinolinic acid, acetoacetic acid ethylmalonic acid, 1,3-dimethyluric acid and alpha-zearalenol in the AD group were remarkably increased compared with the NC group. Among these, 2-methylhippuric acid is normally minor metabolites of fatty acids and is widely used as a biomarker for diseases associated with impaired mitochondrial fatty acid beta-oxidation (Chen et al., 2023). 3-pyridinol induces cell cycle deregulation and apoptosis by shifting redox enzyme activities, particularly affecting reduced and oxidised glutathione levels (Gandhi et al., 2022). Stercobilin acts as a common urobilinoids stemmed from the catabolic conversion of *Lachnospiraceae*, and promotes the production of TNF-α, IL-1β and IL-6 (Sanada et al., 2020). Although sobetirome preferentially accumulates in the liver and is beneficial for reducing the risk of cardiovascular disease by accelerating the depletion of cholesterol, high levels of sobetirome may destroy the heart function (Lammel Lindemann and Webb, 2016). Vanillylmandelic acid, an organic compound involved in various enzymatic reactions, is applied to diagnose neuroblastomas (Beals et al., 2022). Quinolinic acid, produced in the central nervous system, has the ability to promote inflammatory responses by stimulating the N-methyl-D-aspartic acid receptor (Hestad et al., 2022). Acetoacetic acid is commonly found in the liver of patients with metabolic disorders, resulting in excessive fatty acid breakdown and oxidative stress (Shi et al., 2016). Ethylmalonic acid is an organic acid metabolite that destroys succinate and glutamate oxidation, leading to increased mitochondrial permeability (de Moura Alvorcem et al., 2021). 1,3-dimethyluric acid mainly stems from the theophylline metabolism in the liver and is subsequently transferred to the blood and urine, which it can aggravate renal failure (Yu et al., 2002). alpha-zearalenol acts as a mainly liver metabolite that promotes the production of reactive oxygen species by regulating the Bcl-2/Bax pathways in mitochondrion (Lu et al., 2013). These results provided evidence that the changes in gut metabolites in AD patients have the potential capacity to exacerbate state of AD. Although our analysis revealed a significant enrichment of various metabolites in the gut of AD patients, it remains unclear which specific metabolites are responsible for driving the pathogenesis of AD.

## 5 Conclusion

In this study, blood pressure and α-diversity (including the ACE and Chao1 indexes) of the gut microbiota were obvious increased in AD patients. Additionally, pronounced alterations were observed in the composition of the gut microbiota and the profile of gut metabolites, which may be involved in galactose metabolism, caffeine metabolism, and tyrosine metabolism. The above results have remarkable implications for providing the scientific evidence to support the diagnosis of latent AD. While our study provides initial understanding of key themes, the small sample size requires that the results be interpreted with caution.

## Data Availability

The datasets presented in this study can be found in online repositories. The names of the repository/repositories and accession number(s) can be found in the article/**SupplementaryMaterial**.
