# Supplementary material for: Microbiomics and metabolomics explored the characteristics of gut microbiota and metabolites in patients with aortic dissection
Source: Front Cell Infect Microbiol. 2025 Oct 8;15:1677726. doi: 10.3389/fcimb.2025.1677726 (PMC12540387; doi:10.3389/fcimb.2025.1677726)

**Supplementary data**

**Table S1** The relative abundance of gut microbiota at the genus level between the AD and NC groups.

| Alteration  (AD Vs. NC) | Key bacteria |
| --- | --- |
| Up-regulation | *norank_f_[Eubacterium]_coprostanoligenes_group*, *UCG-002*, *Desulfovibrio*, *norank_f_Oscillospiraceae*, *UCG-005*, *Hungatella*, and *NK4A214_group* |
| Down-regulation | *Bifidobacterium* and *[Eubacterium]_eligens_group* |

**Table S2** The alteration in gut metabolites in the AD and NC groups.

| Alteration  (AD Vs. NC) | Key metabolites |
| --- | --- |
| Up-regulation | Adenylosuccinic Acid, 3'-Deoxyderhamnosylmaysin, Apigenin, 7-Di-O-Xyloside, N-Palmitoyl Aspartic Acid, Rubiadinprimeveroside, Multiflorin B, Galactaric Acid, Gentiopicrin, 5-Methoxysalicylic Acid Sulfate, Parishin C, Apigenin 7-Sulfate, Diferuloyl Putrescine, Cgp71422, Genistein 7-Sulfate, (-)-Epigallocatechin 3-Glucuronide, 4-Hydroxytriazolam, Lys-Glu-Asp, Codeine-6-Glucuronide, Glucuronidated Sn-38, Emodin, Ochromycinone, 3-Fucosyllactose, Ala-Ala-Tyr, 4-Methylcatechol 2-Sulfate, Plumieride, Clethodim, Maltotetraose, Malonylgenistin, Isoschaftoside, Demethoxycentaureidin 7-O-Rutinoside, Glu-Phe-Asp, Apigenin 7-Galacturonide, Phloretin Xylosyl-Galactoside, Eriocitrin, Luteolin 7-Sulfate, Sinapic Acid Sulfate, Vanillin-4-Sulfate, Taraxacoside, Baicalin, Stachyose, P-Sulfoxy-Cinnamic Acid, Glycogen, Asn-Pro-Tyr, 5-Hydroxyindoleacetaldehyde, Tetranor 12-Hete, Ophthalmic Acid, Vicenin Iii, Enterolactone 3''-Sulfate, S-Sulfo-L-Cysteine, Homovanillic Acid Sulfate, N,N-Dimethylguanosine, Prenyl Glucoside, Ulifloxacin, Shanzhiside, 3,4-Dihydroxybenzenesulfonic Acid, Raffinose, 4A-Carbinolamine Tetrahydrobiopterin, Cholesterol Glucuronide, Cinnamoylglycine, 5-(2'-Carboxyethyl)-4,6-Dihydroxypicolinate, 2,4-Thiazolidinedicarboxylic Acid, 2-Methyl-, Asp-Tyr-Ile, Ethyl Ferulate, 3-Methyluridine, Cloranolol, Val-Asp-Asp, Spionoside B, 6:3+6O Fatty Acyl, Hexoside, Genisteol 7-Monoglucoside, Isovitexin 2''-O-Rhamnoside, Procyanidin B1, 3-O-Caffeoylquinic Acid Methyl Ester, Taurocholic Acid 3-Sulfate, Tyr-Asn-Ser, 2-Hydroxyfelbamate, Dl-Arginine, Trans-Cinnamoyl-Beta-D-Glucoside, 3'-Benzoylsalicin, Dicoumaroyl Spermidine, Dehydrocurdione, 2-(1-Ethoxyethoxy)Propanoic Acid, 2-Furoic Acid, (+)-8-Acetoxycarvone, Physapubescin, Lactofen, Fexofenadine, 3H-Indole-3-Propanoic Acid,A-Amino-, Val-Thr-Asn, Asp-Asn-Leu, Kelampayoside A, Gly-Ser, N-Acetyl-L-Tryptophan, (E)-2-Glucosyl-3,4',5-Trihydroxystilbene, Lactarorufin B, Tyr-Gly-Ser, O-Methoxyphenyl Sulfate, Met-Asp, N-Lactoylleucine, Implitapide, Ent-Rosuvastatin Lactone, Flavanone Base+3O,C-Hex, Taurine, Temazepam Glucuronide, Asn-Gly-Leu, Sambutoxin, Mytilin A, Ile-Asp-Asp, Sulindac Sulfide, Repaglinide Aromatic Amine, Tryptophyl-Glutamate, Phe-Gln, Manninotriose, 3-Sulfobenzoic Acid, Oxazepam Glucuronide, Leu-Gln, Ponasterone A, Tuberonoid A, Asp Gly Val, Pgf2Alpha Serinol Amide, and Temocapril |
| Down-regulation | 1,4-Cyclohexanedicarboxylic Acid, N-Eicosapentaenoyl Glutamic Acid, Gemopatrilat, Isolimonic Acid Glucoside, 2-Methylhippuric Acid, L-Rhamnulose, Threonylhydroxyproline, Altenusin  3-Pyridinol, 4,6-O-Ethylidene-D-Glucose, Andrographolide  Halometasone, 8-Hydroxy-7-Methylguanine, 8-Angeloylegelolide, Fahfa(2:0/20:0), Melatonin Glucuronide, Citreoviridinol A1, 25-Hydroxyvitamin D3 3-Sulfate Ester, Cucurbic Acid, 5-Hydroxy-3',4',7,8-Tetramethoxyflavone, Gentisic Acid, Haematommic Acid, Ethyl Ester, Daumone, 4-Hydroxybenzyl Alcohol, Baptifoline, Stercobilin, 3,5-Dimethoxycinnamic Acid, Ile-Met, Gibberellin A8, Gly-Gln, Fahfa(2:0/22:0), Abu-Hophe-Oh, Trans-1,2-Dihydrobenzene-1,2-Diol, Masoprocol, Tetrahydro-2,5-Furan-Diacetic Acid, Acetoin, N-Palmitoyl Cysteine, Prostaglandin-C2  Lamiidoside, Triacanthine, Penciclovir, Dl-3,4-Dihydroxyphenyl Glycol, Sobetirome, P-Hydroxyubenimex, Xanthomicrol, Ethyl Beta-D-Fructofuranoside, 2-Hexyl-3-Hydroxypentanedioic Acid, Cis-Resveratrol 3-O-Glucuronide, Mimosine, Prunasin, 3,3'-Dihydroxy-4',5,7-Trimethoxyflavan, 3-Methylorsellinic Acid, 5-Chloro-2'-Deoxyuridine, His-Gly-Gln, Megestrol, 1-(3-Pyridinyl)-1,4-Butanediol, Arabsin, Acifran, Esculetin, Lysyltryptophan, 4',7-Dihydroxy-6-Methoxyisoflavan, (R)-2-Hydroxystearic Acid, Dihydroresveratrol 3-Glucuronide, Methylisopelletierine, Chaetoxanthone B, Beraprost, 2-(2-Furanyl)-3-Piperidinol, 4-Hydroxy-Benzenepropanedioate, Valtrate, (-)-Epigallocatechin 3-(4-Methyl-Gallate), N-Formyl-Met-Leu-Phe, 5'-Hydroxy-3',4',7-Trimethoxyflavan, Ibrutinib, Phe-Phe-Lys, L-Citronellol Glucoside, (Z)-3-Hexenal, Deoxyelephantopin, 1-Cyclohexene-1-Carboxylic Acid, Cyperine, Leucyl-Tryptophan, Vanillylmandelic Acid, Absindiol, Topaquinone, Quinolinic Acid, St(24:2_O4), Cartorimine  Ile-Phe, 2-Methylbenzoic Acid, 3,4,5,6-Tetrahydrohippuric Acid, Cromakalim, 6-(2-Hydroxyethoxy)-6-Oxohexanoic Acid, Tryptophyl-Valine, Gly-Pro-Arg-Pro, 5-(4-Hydroxybenzyl)Thiazolidine-2,4-Dione, Biotripyrrin-B, C75 Trans, 4-Hydroxyretinoic Acid, Dacinostat, 1-Methylxanthine, Acetoacetic Acid, 4,5-Dihydroniveusin A, Ethylmalonic Acid, Cyclo(Leu-Phe), Nopalinic Acid, 1,3-Dimethyluric Acid, Teloxantrone, 6-(4-Phenylpiperazin-1-Yl)Hexanoic Acid, Ile-Ile-Thr, His-Phe-Arg, 4',5,8-Trihydroxyflavanone, 3Beta,6Beta-Dihydroxynortropane, Dihomolinoleic Acid, Frenolicin B, Momilactone A, Menthyl Pyrrolidone Carboxylate, Coniferyl Alcohol, Dodeca-6,9-Dienedioylcarnitine, Alpha-Zearalenol, Mesobilirubinogen, Phyllanthusol B, Zidovudine Glucuronide, Vulgarole, 4-Ethylphenol, Rhamnose, Tilisolol, 2-Methylpyridin-3-Ol, Fahfa(2:0/23:0), Repsox, Tuckolide_ Decarestrictine D, Glucose Butyrate, Isokobusone, Thr-Pro-Tyr, Af Toxin Ii, Fahfa(2:0/24:0), Flavonol 3-O-D-Glucoside, Cyclohexanecarboxylic Acid, 5-Hydroxyflavone, St(24:1_O4_G/18:1), Glyvenol, Demethylvestitol, 2-Acetyl-1-Ethylpyrrole, Dihydroorotic Acid, Il-1R Antagonist, Tyr-Tyr, Nanaomycin, Abu-Gly-Oh, Cassythicine, 4,11,13,15-Tetrahydroridentin B, Lupinic Acid, 2,6-Dimethylaniline, Ubenimex, 3',4'-Dihydroxyacetophenone, 3-Methyl-1-Propylxanthine, 1,7-Dimethyluric Acid, Citrusin I, Pinolidoxin, 4-Nitro-3-(Trifluoromethyl)Phenol, Macrophorin B, 6-Epi-7-Isocucurbic Acid Glucoside, Tyrosyl-Phenylalanine, Orcinol, 5Z-7-Oxozeaenol, Indole-3-Methyl Acetate, Cysteinyl-Methionine, Peliglitazar  Myrianthic Acid, 2,3-Dinor-Txb2, Atorvastatin, 4-(3-Pyridyl)-3-Butenoic Acid, 4-Hydroxynonenal Glutathione, Tyrosyl-Leucine, Cerasinone, Phenylalanyltyrosine, Sedanonic Acid, Fahfa(2:0/24:1), Variabilin, Acetylstrophanthidin, Nepetalactam, 2,3-Dihydroxycarbamazepine, Trp-Val, Obtustyrene, Lithocholic Acid, Pisumionoside, and N-Phenylacetyl Pyroglutamic Acid |

**Figure S1** Image of computed tomography in AD patients.


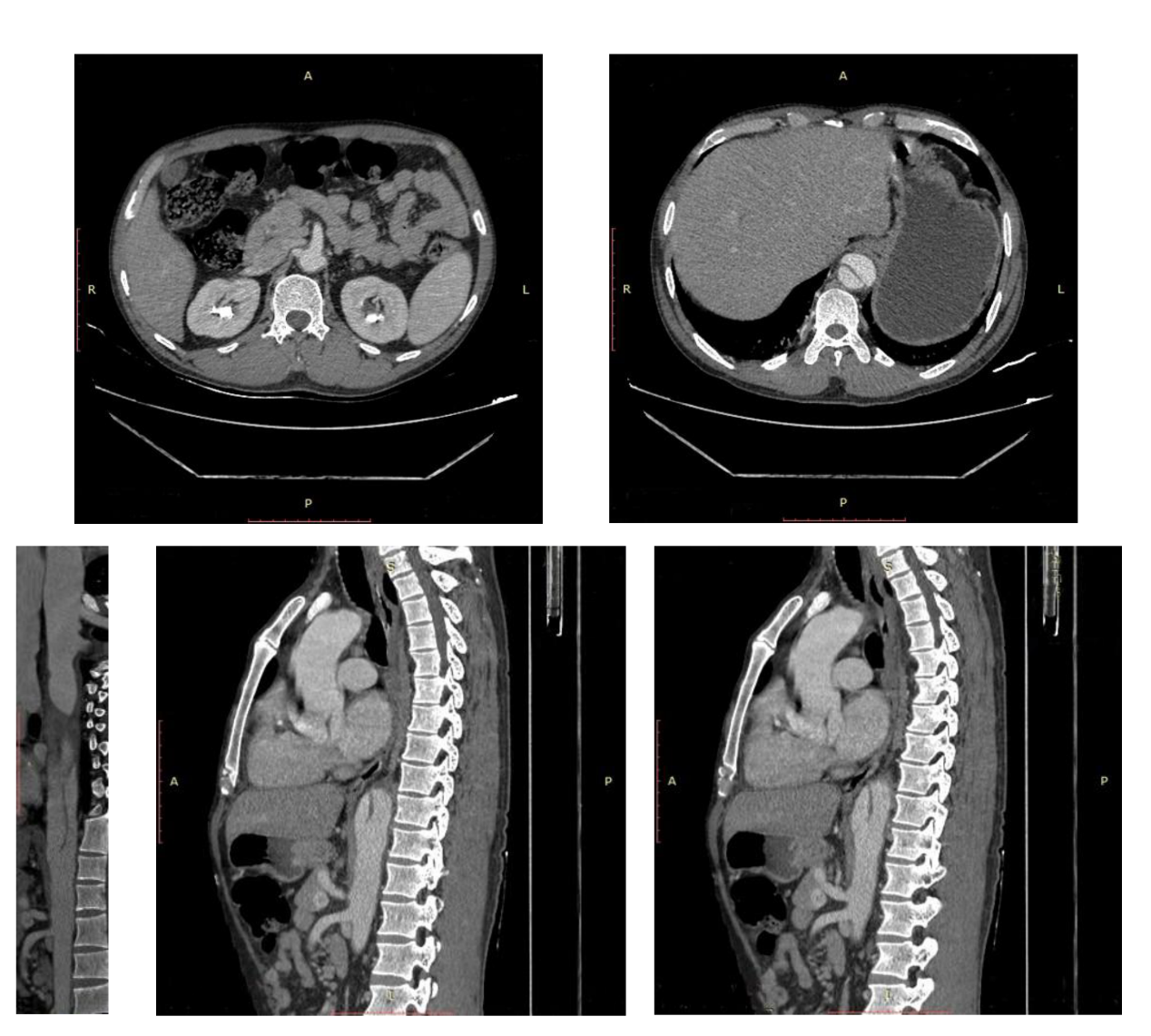

Supplement: Supplementary file 1 [file Table1.docx]
